# Triage for out-of-hours primary care calls: a reliability study of a new French-language algorithm, the SALOMON rule

**DOI:** 10.1080/02813432.2019.1608057

**Published:** 2019-04-29

**Authors:** Edmond Brasseur, Jean-Christophe Servotte, Anne-Francoise Donneau, Samuel Stipulante, Vincent d'Orio, Alexandre Ghuysen

**Affiliations:** a Emergency Department, University Hospital Center of Liege, Liege, Belgium;; b Medical Simulation Center of Liege, University of Liege, Liege, Belgium;; c Public Health Sciences Department, University of Liege, Liege, Belgium;; d Medical Informatics and Biostatistics Department, University of Liege, Belgium;; e Federal Public Health Services, Liege, Belgium

**Keywords:** After-hours care, emergency medical services, primary healthcare, algorithm, telephone, triage nurse

## Abstract

**Introduction:** Triage systems for out-of-hours primary care physician (PCP) calls have been implemented empirically but no triage algorithm has been validated to date. A triage algorithm named SALOMON (Système Algorithmique Liégeois d’Orientation pour la Médecine Omnipraticienne Nocturne) was developed to guide triage nurses. This study assessed the performance of the algorithm using simulated PCP calls.

**Methods:** Ten nurses were involved in 130 simulated PCP call scenarios, allowing the determination of SALOMON’s inter-rater agreement by comparing the actual choices of a specific triage flowchart and the level of care selected as compared with reference assignments. Intra-rater agreement was estimated by comparing triage after training (T1) and 3 to 6 months after SALOMON use in clinical practice (T2).

**Results:** Overall selection of flowcharts was accurate for 94 .1% of scenarios at T1 and 98.7% at T2. Level of triage was adequate for 93.4% of scenarios at T1 and 98.5% at T2. Both flowchart and triage level accuracy improved significantly from T1 to T2 (*p* < 0.0001). SALOMON algorithm use is associated with a 0.97/0.99 sensitivity and 0.97/0.99 specificity, at T1/T2 respectively.

**Conclusions:** Results revealed that using the SALOMON algorithm is valid for out-of-hours PCP calls triage by nurses. The criterion validity of this algorithm should be further evaluated through its implementation in a real life setting.

## Introduction

Over the past decades, primary care facilities in Belgium have faced a dramatic shortage of general practitioners (GP) leading to a major increase in GP workload and over-crowding in emergency departments (ED). This mismatch between the reduced availability of medical resources and the increasing needs of the population, related notably to aging, have led many Western countries to focus on optimising of out-of-hours (OOH) primary care services [1–5].

When managing non-programmed care, patients must find their own way into a sometimes rather complex process of choosing among several options, based on their common sense and expectations. For life-threatening situations, they may call 112, the European emergency number or, in less critical conditions, try to reach a GP or an ED, either by calling or visiting them. However, many concerns have been raised by this decision process because patients’ criteria for the evaluation of the acuity and severity of complaints rarely match healthcare professionals’ opinions, leading to improper use of emergency medical resources and healthcare [6–9].

To ensure patient safety and high-quality healthcare, patients with urgent conditions need to be triaged from those who could wait for a medical consultation at a later date. Consequently, most ED triage scales have been developed to categorize incoming ED patients and prioritize those in need of urgent care over less urgent cases [10–13]. Correspondingly, primary care physician (PCP) triage tools have been developed in The Netherlands [14], Denmark [15], The United Kingdom [16], Norway [17] and Switzerland [6], aiming to get the patient to the right place within a reasonable timeframe.

Organizational models of telephone triage vary in terms of the involvement of physicians [18], nurses or nurse assistants [1,19], or, those based on an empirical or algorithmic frame. Indeed, telephone triage is a rather complex task, lacking visual contact. The patient's ability to communicate symptoms or describe signs makes it challenging, with the risk of under estimating the degree of emergency care required [2,20]. In contrast, many studies [6,19] have demonstrated that training, based on medical knowledge, communication and triage skills offer an opportunity to improve appropriated triage assessment.

Several validated emergency scales dedicated to triage patients at ED admission exist [10–13], however, to our knowledge, no validated telephone triage tools for PCP calls have been reported to date. In order to guide nurse triage PCP out-of-hours calls in Belgium, we developed a specific French-language triage algorithm and named it SALOMON (Système Algorithmique Liégeois d’Orientation pour la Médecine Omnipraticienne Nocturne). This study investigates the criterion validity of the algorithm using simulated clinical case scenarios reproducing day-to-day practice. We also evaluated the feasibility of this procedure with regard to time pressures.

## Material and methods

### The SALOMON algorithm

SALOMON was developed by a group of experts including GPs, emergency physicians and members of the Emergency Medical Services. The methodology employed for the development of the algorithm included the Delphi method [21], and face-to-face meetings with experts. A consensual approach was used aiming to find the most efficient way to help nurses triage and dispatch primary care physicians’ calls, particularly during out-of-hours. The complete algorithm is available at: http://www.chu.ulg.ac.be/jcms/c_1702234/le-projet.

SALOMON is a step by step process guiding nurses’ decisions concerning the level of care needed, and also the most appropriate timing and location. The first step consists of the application of general rules for processing the call, observations and indicators, pain assessment, etc. The second step concerns the circumstantial collection of relevant information. Several specific questions were created to help nurses’ dispatching process, with a specific encoding form. When making the assessment, steps and criteria are used as guidelines rather than absolute rules. Whenever a nurse recognized a life-threatening condition, the call was immediately referred to the 112 dispatching center.

The SALOMON algorithm gathers 53 major flowcharts allowing it to handle most common calls. These flowcharts are identified on the basis of the main complaints, symptoms and available signs, using general and specific discriminators. From the outset, we were careful to use a triage process similar to classical triage systems (Manchester Triage Scale, Emergency Severity Index) and to the Belgian 112 Dispatchers Handbook.

By applying these flowcharts, nurses triage calls and dispatch them into four categories of care (from 1 to 4) corresponding to decreasing levels of urgency and severity:

- SALOMON level 1: Severe: Emergency Medical Services Intervention (EMSI). Triage nurses immediately contact the 112-dispatching center to send emergency medical service to the scene.
- SALOMON level 2: Moderate: Non-urgent Emergency Department Consultation (NEDC). Triage nurse advises patient to attend an ED by their own means or to call an ambulance.
- SALOMON level 3: Minor: Primary Care Physician Home (PCPH). Triage nurse refers the patient to the GP on call.
- SALOMON level 4: Benign: Primary care Physician Delayed visit (PCPD). Triage nurse advises the patient to contact their GP during office hours.

PCP OOH triage nurse training consisted of a 20-hour theoretical teaching course, 4-hours practical skill training and a 4-hour residency in a 112-dispatching center under the supervision of an emergency physician.

### Study settings and population

This study was conducted in two facilities of the University Hospital of Liege: CHU Sart-Tilman and CHU Notre-Dame (CHUNDB), consisting of 622 and 263 beds respectively and an ED census of around 90 000 patients each year. CHU Sart-Tilman is a tertiary care ED located in the suburban area of Liege, while CHU Notre-Dame des Bruyères is a secondary care ED located downtown.

All nurses involved in the study were specialized emergency care nurses with at least 2 years experience. They were also experienced in ED triage of incoming calls through the use of the Echelle Liégeoise d’Index de Sévérité à l’Admission (ELISA) triage scale, as previously described [11]. Participation was on a voluntary basis informed consent was obtained after a comprehensive explanation of the aims of the study.

**Table 1.** Nurses Flowchart selection and SALOMON level determination as compared with reference.

	T1	T2	*p* Value
Flowchart			<0.001*
Correct	1223 (94.1%)	1283 (98.7%)	
Incorrect	77 (5.9%)	17 (1.3%)	
Flowchart and level			
Correct	1164 (89.5%)	1268 (97.5%)	<0.001*
Incorrect	136 (10.5%)	32 (2.5%)	
Level			
Correct	1214 (93.4%)	1280 (98.5%)	<0.001*
Incorrect	86 (6.6%)	20 (1.5%)	
Subcategorization	39 (3%)	7 (0.5%)	<0.001**
Overcategorization	47 (3.6%)	13 (1%)	

*McNemar’s Test; **Test of symmetry.

The ethics committee of the University Hospital of Liege approved the design of the study.

### Study protocol

This prospective study was designed to assess the criterion validity of the SALOMON algorithm. Three emergency physicians and two GP experts developed 130 scenarios derived from real cases. Scenarios design covered the complete range of flowcharts. For each scenario, the team of experts defined which protocol should be selected and which triage level should be attributed to the case. These assignments were considered as gold standard.

The nurses (*n* = 10) were exposed to the 130 randomly simulated calls at two different time points: immediately after the training completion (T1), then three to six months after (T2). During the time interval between T1 and T2, nurses dispatched real patients' phone calls.

The SALOMON algorithm was assessed in simulated PCP calls involving standardized patients. Two amateur actors (one woman and one man) were trained as callers. Simulated calls were audio and video recorded and the recordings further reviewed by an independent blinded rater.

### Measurements

Criterion validity was estimated by studying the correlations between the flowchart selected, the SALOMON level attributed by the nurses, dispatchers and the gold standard. In addition, for each scenario (*n* = 130), the duration of the call was measured.

As a result, data corresponding to 2600 PCP simulated calls were available for analysis.

### Statistical analysis

#### Algorithm and urgency selected

We compared the flowchart selected and SALOMON level attributed by the nurses at T1 and T2 with the gold standard by a McNemar’s Test. Next, we measured over and under classification at each time.

The sensitivity, specificity, and positive predictive values (PPV) and negative predictive values (NPV) of SALOMON were also evaluated. The 4-point urgency scale was divided into high urgency (SALOMON level 1 and 2) and low urgency (SALOMON level 3 and 4). To calculate NPV and PPV, we used prevalence of emergency calls in the scenarios.

#### Duration of the call

Duration of the call was compared between T1 and T2 with two-sided Wilcoxon signed-rank.

## Results

### Population

Ten nurses were recruited on a voluntary basis; 5 males and 5 females. The mean age of nurses was 33.7 ± 8.2 years-old (range: 25–49), with a mean professional experience of 13 ± 4.5 years.

### Algorithm and urgency selected

**Figure 1.**
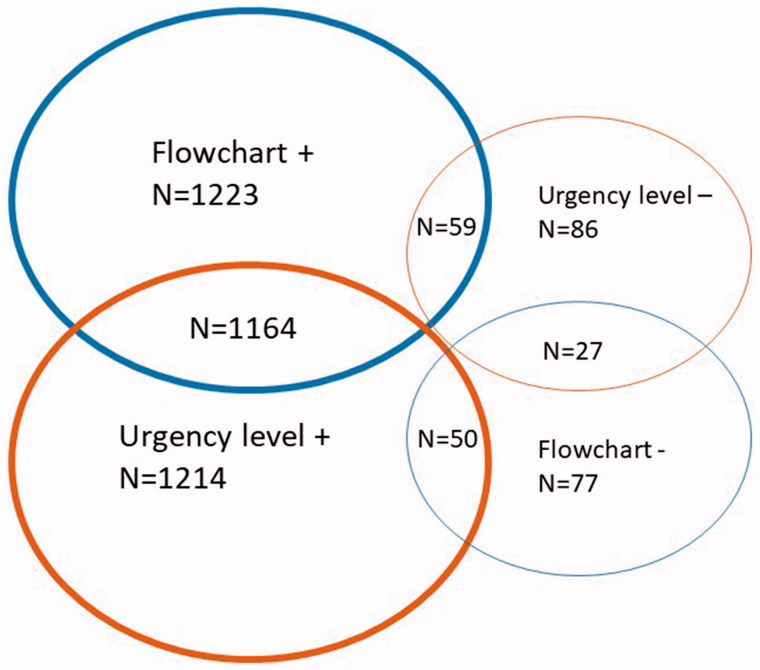
Nurses Flowchart selection and SALOMON level determination as compared with reference at T1.

**Figure 2.**
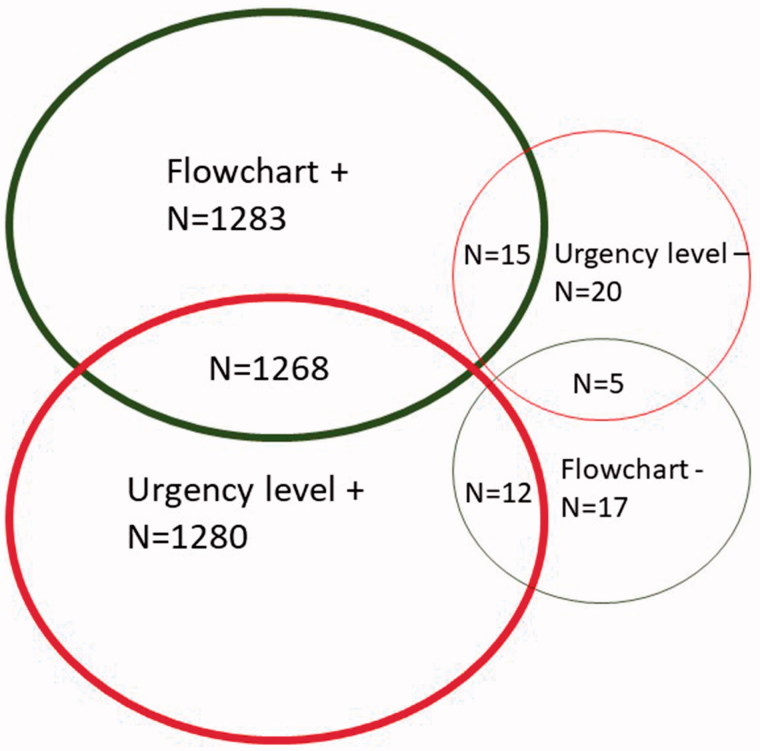
Nurses Flowchart selection and SALOMON level determination as compared with reference at T2.

According to the experts’ triage categorization, 39.2% of the calls (*n* = 510) were referenced as SALOMON level 1, 18.5% (*n* = 240) level 2, 27.7% (*n* = 360) level 3 and 14.6% (*n* = 190) level 4. Figures 1 and 2 summarize flowchart selection and SALOMON level determination at T1 and T2.

Regardless of which flowchart was chosen, nurses’ choice for SALOMON level matched the reference in 93.4% of the cases at T1 and 98.5% at T2. The improvement between T1 and T2 in level determination was found to be statistically significant (*p* < 0.001) (Table 1).

Comparison of the algorithm selected between nurses and experts revealed a 94.1% agreement at T1 and 98.7% at T2. The difference between T1 and T2 was statistically significant (*p* < 0.001).

Both flowchart selection and SALOMON level determination matched the reference in 89.5% of scenarios at T1 and 97.5% at T2 (*p* < 0.0001).

An incorrect selection of the level of care at T1 was found to lead to 3% (*n* = 39) subcategorization and 3.6% (*n* = 47) overcategorization, but only 0.5% of subcategorization (*n* = 7) and 1% of overcategorization (*n* = 13) at T2. The evolution was statistically significant (*p* < 0.001).

The sensitivity of the SALOMON algorithm reached 0.97 (95% CI = 0.96–0.98) and 0.99 (95% CI = 0.98–1) at T1 and T2 respectively. The specificity was 0.97 (95% CI = 0.95–0.98) and 0.99 (95% CI = 0.98–1) for T1 and T2 respectively. In this study, the prevalence of urgency calls was of 57.69%. The use of the SALOMON algorithm was associated, at T1 and T2, with a PPV of 0.97 (95% CI = 0.96–0.98) and 1 (95% CI = 0.99–1) and a NPV of 0.96 (95% CI = 0.94–0.97) and 0.99 (95% CI = 0.98–1), respectively.

### Duration of the call

At T1, median call time was 41 seconds with an interquartile range (IQR; Q1-Q3) of 30–60 seconds. It decreased to 33 seconds (IQR: 25–43) at T2. The duration of the call was significantly reduced in T2 as compared with T1 (*p* < 0.001).

## Discussion

The aim of this validation study was to evaluate the effectiveness of a specific French-language triage algorithm called SALOMON to guide nurse triage PCP out-of-hours calls. The scientific literature lacks evidence for gold standard PCP triage [22,23]. Common criteria used to assess triage protocols involve validity and simplicity. As for validity, which refers to the degree of agreement between instrument’s users and a gold standard [24], we used a team of experts classification as gold standard.

Our results demonstrated a high degree of agreement between nurses and the gold standard. We found strong agreement for the flowchart and the urgency selection, which improved after training. The SALOMON algorithm allowed nurses to triage PCP calls efficiently and rapidly from the first triage attempts after initial training. Sensitivity and sensibility are very high as well as PPV and NPV. Moreover, SALOMON has been found to be easy to teach with the planned program that included 20-hour theoretical teaching, 4-hour practical skills training and 4-hour residency in the 112-dispatching center under the supervision of an emergency physician.

Telephone triage is the core of the SALOMON algorithm. Triage nurses face the difficult task of assessing patients without visual contact. In such circumstances, previous studies have demonstrated an average error rate of around 10% related to an underestimation of the level of urgency [2,20]. Under-triage is a critical issue because it can compromise patient safety particularly for EMSI category. In the present study, we found an under-categorization rate of 3% at T1 and 0.5% after three to six months of SALOMON utilization. Over-estimating the urgency of cases can also compromise patient safety due to improper, misallocated resources leading to poor distribution of workload and impaired efficiency [2]. This was also found to be a rare occurrence using SALOMON algorithm.

An interesting finding was that even if the triage nurse selected a different flowchart from reference, the urgency level determined was kept in agreement with the gold standard.

Based on the results, we feel that the SALOMON algorithm has the potential to offer a safe and simple process for ED nurse triage out-of-hours primary care calls. Nevertheless, our study does have limitations.

First, we collected our research data in two different emergency departments, but under the management of the same institution. This could limit the capacity to generalize results to other triage primary care call centers and facilities because nurses had former experience in ED triage. Second, the small number of participants (triage nurses) included (*n* = 10) in combination with two actors is another limitation of the study. The nurses not only participated on a voluntary basis in the research but also were highly motivated by the study, triage, and training. It would be important to evaluate if nurses with a less favourable profile could obtain the same results. Further studies should increase and diversify the number of participants and the number of actors.

Participating nurses may have recognized the simulated calls, which may have contributed to the improvement of the duration of the calls. Furthermore, the use of simulation meant that we could not test the algorithm against all call types or circumstances which, in the ED settings might be infinite. However, the use of simulation instead of real calls for assessment was chosen in the interests of patient safety with the idea of: “Never the first time on the patient”. Indeed, in designing our experiment, we considered it unethical to triage patients before testing and validating the scale. According to Roter et al. [25], such a method is valid to assess competences. Nevertheless this study is a criterion-based validity study made under simulation environment, using a reference standard based on consensual clinical experts’ opinion. Whether or not this standard still holds true in real life should be further determined in real life conditions.

## Conclusion

In summary, the needs for the development of a specific, French-language triage algorithm to guide nurse triage PCP out-of-hours calls led us to design the present study revealing that the SALOMON algorithm offers a robust, valid, simple and easy to learn tool to help these nurses triage primary care physicians phone calls.

Our study shows a high sensitivity and specificity of the tool. Of utmost importance is that this tool provides a very safe system for triaging these calls, the risks for under- and over-triage appear extremely limited in simulation.

Further investigations are needed now to assess the impact of the use of this algorithm in clinical settings.
